# How mindfulness-based training improves stress-related health: a selective review of randomized clinical trials comparing psychological mechanisms of action

**DOI:** 10.3389/fendo.2025.1415081

**Published:** 2025-08-04

**Authors:** Lara M. C. Puhlmann, Veronika Engert

**Affiliations:** ^1^ Leibniz Institute for Resilience Research, Mainz, Germany; ^2^ Max Planck Institute for Human Cognitive and Brain Sciences, Leipzig, Germany; ^3^ Clinical Psychology and Behavioural Neuroscience, Faculty of Psychology, Technische Universität Dresden, Dresden, Germany; ^4^ Institute for Psychosocial Medicine, Psychotherapy and Psychooncology, Jena University Hospital, Friedrich-Schiller University, Jena, Germany; ^5^ Center for Intervention and Research on Adaptive And Maladaptive Brain Circuits Underlying Mental Health (C-I-R-C), Halle-Jena-Magdeburg, Germany

**Keywords:** stress-related health, cortisol, sympathetic-adrenomedullary system (SAM), mindfulness, monitoring, acceptance, randomized controlled trials, training-specific effects

## Abstract

Mindfulness-based interventions (MBIs) have been shown to reduce both subjective experiences and physiological markers of stress, a central pathway to improving health and wellbeing. Yet, understanding of the causal mechanism through which MBIs affect stress-related health outcomes remains poor. Most MBIs rely on training programs that simultaneously target multiple and distinct mental processes, hampering mechanistic conclusions. Addressing this shortcoming, the present selective review provides an overview of randomized controlled trials (RCTs) that directly contrast the effects of distinct components of mindfulness on stress-related health. We examine two comparative frameworks, the prominent Monitor and Acceptance Theory (MAT) and the ReSource training program, an intervention protocol designed to disentangle mindfulness components in a large-scale mental training project. We focus on how a) attention monitoring and b) experiential acceptance skills affect the stress-related outcomes assessed. These include subjective-psychological stress and affect, and physiological stress and stress-related health markers (e.g., activity of the autonomic nervous system, the hypothalamic-pituitary-adrenal axis and proinflammatory activity), each in two different states of the stress system: acutely stressful challenges and more long-term basal functioning. In line with MAT, we find that monitoring needs to be coupled with acceptance for beneficial effects on stress-related physiological activity in states of acute challenge. In basal states, however, physiological stress activity can be buffered by monitoring alone, especially if practiced for longer duration. We suggest that when dealing with basal longer-term stress, monitoring allows individuals to use coping mechanisms other than acceptance, such as social support. Subjective-psychological stress and affect were mostly assessed in basal states and show either non-specific effects after all types of training, or are most affected by combined monitoring and acceptance. Our work highlights the need to evaluate different training mechanisms in relation to stress-specific states (herein, basal versus challenge) and outcomes (herein, subjective-psychological versus physiological) in order to better understand mindfulness mechanisms of action.

## 1 Introduction

The experience of stress plays a crucial role in the development and maintenance of both mental disorders and physical health conditions (1, 2). Given the globally rising prevalence of mental health issues (3), one particularly promising healthcare approach involves the reduction of transdiagnostic risk factors, such as the psycho-physiological stress load, for the purpose of prevention (4). Mindfulness-based interventions (MBIs) have become one of the most popular (5) and widely studied (6) interventions for stress reduction and promotion of mental and physical wellbeing. The mechanisms underlying mindfulness training effects, however, remain surprisingly poorly understood, hampering advancement of the field. Research into MBI wellbeing effects for generally healthy adults can inform underlying mechanisms, potential for preventive application, as well as efficacy in the general population. Here, we aim to further this knowledge through a selective review of randomized controlled trials (RCTs) that contrast distinct mindfulness components with regard to their effects on stress-related health outcomes (termed “comparative RCTs” in the following), ranging from subjective-psychological stress and affect, to physiological stress and stress-related health markers.

### 1.1 MBI efficacy for stress-related health outcomes

Mindfulness is a notoriously elusive concept, and a truly unifying definition has not been achieved (7). Building on a prominent definition by Jon Kabat-Zinn (8), mindfulness is operationalized as a particular way of focusing attention characterized by two key features: First, self-regulation of attention towards the present moment and ongoing experiences, which involves attentional monitoring and control. Second, the adoption of a particular orientation to experience characterized by equanimity and acceptance toward each moment of one’s experience (9). Relatedly, “Mindfulness-based interventions” (MBIs) refer to intervention programs that involve repeated training in mindfulness meditation practices (10), or following the above, the conscious cultivation of the mindfulness elements present-moment attention and experiential acceptance (cf. 9).

Over the past three decades, research into the benefits of MBIs has accumulated promising evidence for their capacity to improve health and wellbeing. Recent carefully conducted systematic reviews and meta-analyses identify robust evidence that MBIs improve subjective wellbeing, including perceived stress, depression and anxiety, in both clinical (11, 12) and non-clinical populations (11, 13, 14). Evidence is strongest for at-risk participants, such as those with chronic physical health problems (15, 16), and compared to taking no action (11, 17).

Reductions in subjective-psychological stress as one central aspect of wellbeing are among the best-documented health-related outcomes of contemplative practices for healthy adults to date [e.g., (14, 18)]. This psychological stress load is tightly coupled with activity of the hypothalamic-pituitary-adrenal (HPA) and sympathetic-adreno-medullar (SAM) axes, our two main stress systems [although this relationship is not always straightforward in acute (19) or basal states (20)]. And while mounting an appropriate stress response is necessary during acute threat, prolonged HPA activity leads to health deterioration, reflected, for example, in chronic inflammation and general accumulation of allostatic load (21, 22). Therefore, in the general healthy population, lower stress-induced and overall secretion of the HPA axis end-product cortisol are considered more adaptive^1^, and used to quantify physiological stress-reduction after MBIs. Current evidence for such reduced cortisol secretion is mixed, but increasingly promising [e.g., after acute challenge: (26); in basal state: e.g., (27–29)], particularly for non-clinical at-risk populations (16). Reduced physiological stress load has been proposed as a key pathway through which MBIs improve downstream health and wellbeing (30, 31), making physiological stress indices particularly important endpoints of MBIs.

### 1.2 Mechanisms of mindfulness: current issues

Despite advancements in research synthesis, reviews point out the heterogeneity of study designs and respective wellbeing outcomes [see e.g., (13, 17)], also highlighting that improvements in study quality have been only modest over the years (32, 33). Central to this issue is the inconsistency and often broad nature of intervention protocols, leading to a lack of knowledge about unifying mechanisms and moderators of MBI effects. Typically, interventions simultaneously target multiple and distinct psychological processes, roughly categorizable into attention-, affect- and cognition-based. Mechanistic considerations are predominantly based on correlational studies (34, 35). As such, there is limited evidence directly informing the causal pathways by which MBIs may produce their effects in general, and impact stress-related health outcomes in particular (13, 33, 36, 37).

Understanding different mechanisms of action or “active ingredients” of interventions would form the basis for designing future interventions more effectively and personalizing meditation programs to different populations, contexts and needs. Yet, few selected RCTs have begun to directly compare different MBI components with respect to health and wellbeing outcomes against each other in comparative MBIs. The most notable effort in this context is made by Lindsay and Creswell’s Monitor and Acceptance Theory (MAT), in which the authors conceptualize mindfulness capacities in terms of monitoring and acceptance (31, 38), which they investigated in two dismantling RCTs to date (39). Similarly, the ReSource Project is a multi-method longitudinal RCT that partialized contemplative mental training techniques into the three broad domains of attention, socio-affective and socio-cognitive skills, and tested their distinct effects on a broad range of outcome measures (37, 40). With their randomized and comparative designs, these investigations provide unique assets for understanding MBI effects and relative effectiveness of different mindfulness facets. A first review of MAT dismantling RCTs supports MAT predictions in that acceptance is a critical component for MBI effects on stress sensitivity, positive emotion, and social relationship outcomes (39).

## 2 The current review

With the present work, we aim to advance the causal understanding of MBI training mechanisms through a selective review of randomized controlled trials (RCTs) that contrast distinct mindfulness components with regard to their effects on stress-related health outcomes (termed “comparative RCTs”). We begin by giving an overview of four key accounts that distinguish different mindfulness-based training mechanisms. We then outline and discuss the results of the above comparative intervention protocols, MAT and ReSource. Owing to the multifaceted nature of stress and its effectors (41), we subdivide findings into states of acute challenge to the stress system and basal states. Because we evaluate patterns of identified training effects, we focus only on positive findings (null findings of the respective studies are summarized in the **Supplementary Table S1**).

Health outcomes of different types of MBI programs (rather than mindfulness components) [e.g., mindfulness-based stress reduction (MBSR (42); compassion-focused therapy (43)] have certainly also been tested in a multitude of studies comparing their effects to control groups. As these studies lack mechanistic comparisons of different mindfulness-based practices, they are not considered in this review. Excellent reviews and meta-analyses can be found elsewhere (13, 16, 17, 44). Moreover, **Box 1** gives a short summary of studies investigating the differential relationships of monitoring and/or acceptance skills with stress- and health-related outcomes outside of training intervention designs, in correlational studies or after single session inductions, again grouped by challenge and basal states [see also (38, 39)].

Box 1 **Results from correlational studies and single-session inductions.**

For challenge states, results from correlational and single-session inductions clearly highlight differential effects of monitoring versus acceptance trait expressions or instructions. Findings affirm the possibility of stress exacerbation if only monitoring is practiced (see Creswell & Lindsay, MAT), revealing evidence for relatively increased stress reactivity with higher trait monitoring skills (45), stronger threat responses after awareness manipulation (46), and impaired stress-induced cognitive performance after one session of breath awareness (47). Additionally, high trait monitoring/low acceptance skills were linked to a flatter cortisol awakening response (CAR), and higher trait acceptance/low monitoring skills were linked to steeper CAR (48). While this is contrary to what we find in ReSource [higher CAR after monitoring (Presence), and lower CAR after monitoring and acceptance (Presence and Affect) or acceptance (Affect) training alone; 49; see also Section 3 Overview of key accounts distinguishing mindfulness-related mental processes], the acceptance-specific outcomes were interpreted as adaptive in both instances. This inconsistency can be attributed to distinct perspectives on the CAR in stress research. Much like for the acute stress response, insufficient rises in awakening cortisol would leave an individual unprepared for the challenges of the upcoming day (50, 51). Yet, repeatedly very high CARs may accumulate to allostatic load over time. To that effect, an increased CAR has been linked to job and general life stress (52), depression (53), and borderline personality disorder (54).

Studies correlating monitoring and acceptance abilities in meditation-naïve samples with long-term basal outcomes mostly focused on participant self-reports, and converge well with MAT assumptions. Thus, emotional and health-related symptoms in daily life showed a differentiable pattern with mostly positive outcomes only if monitoring was associated with high acceptance ability. When taken by itself, the ability to monitor present-moment experiences was linked to maladaptive emotional symptoms (55–58). Additionally, lower acceptance alone, independent of monitoring skills, was linked to self-reports of higher chronic stress (59).

Trait monitoring skills alone have also been considered a correlate of *positive* affectivity in some studies (58, 60–62). With the authors of MAT suggesting that high monitoring ability enhances attention to both negative and positive cues, the everyday life context should allow for all possible outcomes, depending on whether negative or positive stimuli dominate in an individual’s life. In other words, heightened monitoring of predominantly positive experiences may be sufficient to intensify positive outcomes, and should not be considered a contradiction to MAT (38, 39).

Again confirming MAT, work testing diurnal cortisol regulation as an indicator of the long-term physiological stress load showed that a self-report profile of high monitoring or one of high monitoring and low acceptance skills was linked to flatter diurnal cortisol slopes (48, 63). Low monitoring and high acceptance or high acceptance skills were linked to sharper cortisol decreases in the evening (48) and steeper diurnal cortisol slopes (63). Steepness in diurnal cortisol slopes is considered an indicator of good mental and physical health (64).

## 3 Overview of key accounts distinguishing mindfulness-related mental processes

Theoretical accounts of the last decade have proposed comprehensive taxonomies that distinguish central elements of mindfulness-based and contemplative practices, broadly based on the involved practice types, underlying cognitive and affective processes and anticipated outcomes (34, 65–68). In the following, we outline four influential frameworks.

### 3.1 Monitor and acceptance theory

The Monitor and Acceptance Theory (MAT; (38) builds on attention monitoring and experiential acceptance as key components of mindfulness (9), with a particular focus on mechanisms driving stress reduction and related health outcomes (30, 31). In MAT, Lindsay and Creswell define attention monitoring as the “ongoing awareness of present-moment sensory and perceptual experiences [ … ] [which] relies on selective and executive attention networks”. Experiential acceptance is defined as a “mental attitude of non-judgment, openness and receptivity, and equanimity toward internal and external experiences”. Thus, acceptance is used in the sense of an umbrella term that encompasses a range of acceptance-related constructs [(38), p. 50]. MAT treats monitoring and acceptance as dissociable skills, and suggests that it is their combination that builds the active mechanisms for mindfulness training effects.

According to MAT, cultivation of attention monitoring will boost awareness of present-moment experience, regardless of whether that experience is positive, negative, or neutral. It will consequently improve cognitive – but not affective – outcomes (38). Given that monitoring skills are likely to develop before acceptance skills (69, 70), initial emotional agitation and symptom exacerbation may happen as monitoring is practiced. Through cultivation of acceptance, however, individuals are predicted to deal with their emotional states more effectively, building on the idea that acceptance skills are central tools of emotion regulation. Specifically, a mental stance of acceptance should modify the relationship with all monitored experiences: negative ones become less potent, neutral ones a source of rest or even pleasure, while positive experiences can be relished. This unified framework has been investigated in two dismantling trials of training monitoring alone (Monitor Only) versus training monitoring plus acceptance (Monitor + Accept) (71–73).

### 3.2 ReSource

The ReSource Program presents a conceptual training framework that targets attentional, affective and cognitive capacities in a comparative intervention design (37, 40). It categorizes meditation and other contemplative techniques into three modules termed Presence, Affect and Perspective. While the ReSource Program goes beyond the training of monitoring and acceptance alone, its modules *do* differentially target attention monitoring and acceptance skills: The core processes cultivated in the Presence module are attention and interoceptive body awareness, thus mapping onto MAT’s Monitor training. The Affect module targets social emotions such as compassion, loving kindness and gratitude, also aiming to enhance prosocial motivation, and includes a unique dyadic training that is focused on the acceptance of difficult emotions (74). This module subsumes, but goes beyond MAT’s Accept training. The Perspective module focuses on meta-cognition and perspective-taking on self and others, with the Perspective dyad honing in on perspective-taking on different internal aspects of the self. This module shares no common mechanisms with monitoring and acceptance as targeted in MAT.

The ReSource Project investigated the potentially differential effects of these modules on a variety of psychosocial and health-related endpoints in a large-scale longitudinal investigation (see **Box 2**). Its outcomes can provide evidence for or against differentiable effects of training monitoring and acceptance, as well as MAT’s specific predictions. In support of the above mapping to MAT, Presence training was found to increase present-moment-focus and body-awareness, counteracting distraction through thoughts (74–76), while Affect lead to greater use of acceptance as an emotion regulation strategy, and reduced maladaptive avoidant strategies (75).

Box 2 **Design of the ReSource Project.**

The ReSource Project (37) is a multimodal longitudinal mental training study realized with N=332 healthy, meditation-naïve male and female participants. Designed as a randomized clinical trial, participants were randomly allocated to one of three training cohorts (TC1, TC2, TC3) completing the training modules (Presence, Affect, Perspective) in different orders, or to a passive retest control cohort (RCC). TC1 and TC2 both started with the attention-based Presence module, followed by the socio-affective Affect and socio-cognitive Perspective modules in reverse order. TC3 only attended the 3-month Affect module, thus allowing to isolate its specific effects. Participants were tested on psychosocial and health-related endpoints at baseline (T0) and after each 3-month training module (T1, T2, T3). Of all the ReSource data assessments, only acute stress testing was realized in a cross-sectional design.

### 3.3 Attentional, constructive and deconstructive families

Contemplative practices and interventions frequently extend beyond training mindfulness as monitoring and acceptance. Conceptualizing the consequently broader mechanisms is a challenging and incomplete endeavor. In one influential account, Dahl and colleagues (65) propose an overarching classification system of different meditation styles into attentional, constructive and deconstructive families - which arguably still subsume monitoring and acceptance as essential and differentiable mechanisms of action (see **Box 3**). While well described, this model has not been translated to a comparative study design.

BOX 3 **Classification of attentional, constructive and deconstructive practices.**

Dahl et al. (65) propose an overarching classification system categorizing different meditation styles based on their cognitive mechanisms into attentional, constructive and deconstructive families. The attentional family targets attention regulation and meta-awareness through the training of attention orientation and openness, monitoring, and detecting and disengaging from distractors. All practices falling under this family should raise practitioners’ awareness of their own thinking, feeling and perceiving. Attention monitoring as understood in the two-component mindfulness model (9), and as adopted by MAT and the trainings of the ReSource Presence module, can be grouped into this family.

The constructive family targets new perspective taking and reappraisal of thoughts and emotions by systematically altering their content. It comprises practices aiming to replace maladaptive beliefs about the self with more adaptive ones. Techniques used to accomplish these aims are, for example, the cultivation of patience and equanimity, of kindness and compassion, and a reorientation of the mind towards true meaning. MAT acceptance, ReSource Affect and the majority of ReSource Perspective training could be categorized within the constructive family.

Practices falling under the deconstructive family aim to undo maladaptive cognitive patterns. Techniques toward this aim are to explore the dynamics of cognition, emotion and perception, thus generating insight into one’s internal models. A mechanism that is central herein is self-inquiry, that is, “the process of investigating the dynamics and nature of conscious experience” [(65), p. 519], often involving both the identification and the questioning of one’s assumptions. Neither attention monitoring, acceptance, nor the techniques of the Affect module translate to the deconstructive family. Specific techniques of the ReSource Perspective module (e.g., training to view events from different internal aspects of the self in the context of the Perspective dyad) could be categorized here. This conceptual model by Dahl and colleagues provides a uniquely non-reductionist concept of mindfulness- and meditation-based mechanisms.

### 3.4 Health-related and mediating mental processes targeted by MBIs

In a less formalized framework, a considerable body of empirical research has also examined mindfulness states and MBIs regarding their neurobiological correlates (77, 78) and putative emotional and cognitive training outcomes and mediators (34, 35). While predominantly correlational, this work has implicated various mental processes as mechanisms of MBI effects, including increased acceptance, emotion regulation, self-compassion, social connectedness, and positive emotions, as well as decreased rumination and negative emotions—processes that may all contribute to MBI health benefits (35, 75, 76, 79). It has been proposed that four key mechanisms drive these cognitive-affective changes: improved a) attention regulation, b) body awareness, c) emotion regulation, and d) self-referential processing (leading to change in perspective on the self) (34, 78, 80).

Compared to other accounts, this approach offers a somewhat more generalizable model of MBI effects that can be integrated with other psychological health research. Again, the proposed mechanisms broadly map onto MAT and ReSource training: Attention regulation and body awareness are mostly targeted in Monitor (MAT) and Presence (ReSource) training. Emotion regulation specifically via acceptance is most directly trained in Acceptance (MAT) and Affect (ReSouce) [although cognitive emotion regulation is also cultivated in ReSource Perspective training; (75)]. Finally, mechanisms for improved self-referential processing are harder to isolate, but most clearly targeted in the combination of Affect and Perspective training (ReSource), and, to a lesser degree, in MAT Accept training, potentially via (self-)acceptance (although this cannot be clearly differentiated).

## 4 Overview of comparative RCT effects on stress-related markers

**Figure 1:**
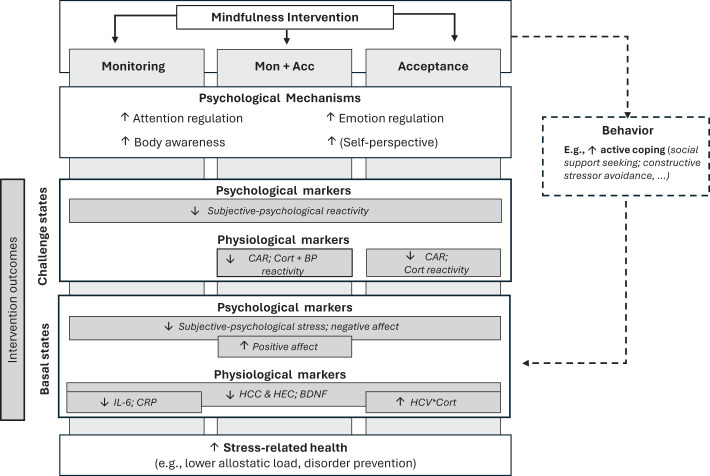
Summary of reviewed study outcomes and potential underlying pathways. By training monitoring and acceptance skills, mindfulness-based interventions can reduce psycho-physiological stress markers and improve stress-related health. This involves change in key psychological mechanisms, and putatively in behavior. Intervention outcomes (grey rectangles) vary by intervention type (monitoring, acceptance or their combination [Mon + Acc]), state of the stress system (basal or challenge) and targeted outcome measure (subjective-psychological stress and affect, or physiological stress and stress-related health markers). See also Creswell et al. (30) for a summary of mindfulness training mechanisms for physical health outcomes. Mon, Monitoring; Acc, Acceptance; CAR, cortisol awakening response; BP reactivity, Blood pressure reactivity; IL-6; interleukin-6; CRP, C-reactive protein; HCC, hair cortisol concentration; HEC, hair cortisone concentration; BDNF, Brain-derived neurotrophic factor; HCV, hippocampal volume.

Below, we summarize the reviewed evidence separately for each protocol (ReSource versus MAT) and stress state (challenge versus basal). In detail, we examine evidence for the existence of differential training effects on subjective-psychological stress and affect, as well as physiological stress and stress-related health markers for monitoring- versus acceptance-related training in particular, and for the specific predictions of MAT (38, 39). Following the above-described mapping, we consider the hypothesized effects of monitoring, acceptance and their combination supported in ReSource data if there is matching evidence from Presence training, Affect training, or their combination, (e.g., an effect of combined monitoring and acceptance would be supported by an effect of combined Presence and Affect training in ReSource). Results are summarized in **Table 1** and **Figure 1**.

**Table 1:** Summary of positive results stemming from randomized controlled studies comparing different mechanisms of mindfulness-based interventions.

Challenge states					
RCT	Study	Method	Finding		
			Stress marker (direction of change)		Effect of practice type
ReSource (N=332)	(26)	3–6 months intervention TSST	Subjective-psychological reactivity (STAI)	↓	All practice (vs. NT)
			Cortisol reactivity	↓	Aff; Pres+Aff; Pres+Persp (vs. NT; Pres)
	(49)	3–9 months intervention Daily life sampling	Cortisol awakening response	↓	Aff; Pres+Aff (vs. NT; Pres; Pres+Persp)
				↑	Pres (vs. NT)
Dismantling Trial 2 (N=153)	(73)	2-week intervention TSST-like	Cortisol reactivity	↓	Mon+Acc (vs. AC; Mon)
			Blood pressure reactivity	↓	

Basal states					
ReSource (N=332)	(81)	3–9 months intervention MRI, daily life sampling	Hippocampal volume ^*^ total daily cortisol output	↑^*^↓	Aff (vs. all other practice)
	(28)	3–9 months intervention Hair sampling, self-report questionnaires	Hair cortisol and cortisone	↓	All practice with time (vs. NT)
			Subjective-psychological stress (PSS)	↓	Persp (vs. NT; Pres+Aff)
	(82)	3–9 months intervention Blood sampling	Brain-derived neurotrophic factor ^*^ hair cortisol	↑^*^↓	All practice with time (no NT included)
			Brain-derived neurotrophic factor ^*^ hippocampal volume	↑^*^↑	
	(83)	3–9 months intervention Blood sampling	Interleukin-6	↓	Pres (vs. NT; Aff)
			C-reactive protein (men)	↓	Pres (vs. NT; Aff; Persp)
Dismantling Trial 1 (N=137)	(71)	8 weeks intervention Daily life sampling	Subjective-psychological stress (Likert scale from 1-7)	↓	Mon+Acc (vs. NT; Mon)
			Subjective-psychological stress (Likert scale from 1-7)	↓	All practice and NT
	(72)	8 weeks intervention Daily life sampling	Positive affect (Likert scale from 1-7)	↑	Mon+Acc (vs. NT; Mon)
			Negative affect (Likert scale from 1-7)	↓	All practice (vs. NT)
Dismantling Trial 2 (N=153)		2 weeks intervention Daily life sampling	Positive affect (Likert scale from 1-7)	↑	Mon+Acc (vs. AC; Mon)
			Negative affect (Likert scale from 1-7)	↓	All practice and AC

AC, active control; Acc, Acceptance; Aff, Affect; Mon, Monitor; MRI, Magnetic Resonance Imaging; NT, no training; Persp, Perspective; Pres, Presence; PSS, Perceived Stress Scale (84); STAI, State Trait Anxiety Inventory (85); RCT, randomized controlled trial; TSST, Trier Social Stress Test.

Blue: Differential training effects confirming MAT (e.g., Monitor alone does not reduce or else increases stress; Monitor + Acceptance reduces stress more than Monitor alone) or going beyond MAT (Acceptance/Affect alone reduces stress more than Monitor alone).

Yellow: No differential training effects contradicting MAT; Differential training effects contradicting MAT (e.g., Monitor/Presence alone reduces stress).

↑, increased after training; ↓, decreased after training.

### 4.1 Challenge states

Most prominently, states of acute challenge to the stress system are measured in the context of standardized laboratory paradigms such as the Trier Social Stress Test [TSST; (86)], which reliably trigger the activation of SAM and the HPA axes (1). Additionally, the cortisol awakening response (CAR) as one distinct aspect of the cortisol diurnal rhythm (87) can be understood as a response to challenge (88) – the difference being that the CAR is a response to an internal challenge (waking up with the anticipated demands of the upcoming day on one’s mind; 51) rather than an external one.

#### 4.1.1 ReSource

ReSource participants attended a TSST after training either the Presence, Affect, the socio-cognitive Perspective module, or their combinations [details see (26)]. All training equally reduced the self-reported psychosocial stress response when compared to no training. With respect to a physiological correlate of stress, acute cortisol release was reduced only after either Affect training alone, combined Presence and Affect, or combined Presence and Perspective training. The 3-month attention-based Presence module alone had no effect on the cortisol stress response (26).

In another study with mostly the same pool of participants, we found a specific effect of Affect training on the CAR (49). Results revealed a stable reduction in CAR after the Affect Module, whether tested after only 3-month Affect training, after 6-month Presence and Affect training, or after 9-month Presence, Perspective and Affect training. By contrast, 3-month attention-based Presence training alone consistently led to a CAR increase.

Evidence from these two studies confirms the presence of differential effects of monitoring and acceptance-related, as well as socio-cognitive practice on cortisol release. They further align with MAT’s specific notion that monitoring (Presence) practice alone is insufficient to reduce, or may even increase, stress reactivity (26, 49; see also **Table 1**), at least with regard to physiological outcomes, while highlighting unique effects of acceptance (Affect) and socio-cognitive training alone (26).

#### 4.1.2 MAT

In a 2-week smartphone-based dismantling RCT in stressed community adults, the MAT authors compared the effects of standard monitoring and acceptance training with a structurally matched training focusing only on monitoring (73). Monitor and Accept training decreased cortisol and blood pressure reactivity to acute psychosocial stress (modified TSST) compared to Monitor Only and active control trainings. There was no indication of Monitor training alone yielding worse stress outcomes than the active control intervention.

### 4.2 Basal (longer-term) stress

Just as for challenge states, there are different approaches to probe the medium to long-term stress load and stress-associated health. For HPA axis-based measures, most frequently, the diurnal cortisol profile is assessed. While the CAR is a dynamic facet distinct from the cortisol circadian rhythm (87), total diurnal cortisol output and the cortisol slope over the course of the day are considered indices of mid-term HPA axis regulation. Higher total output (reflecting cumulative tissue exposure to cortisol) and flatter slopes are interpreted as indices of chronic stress (89). Moreover, hair cortisol and cortisone levels are indices of the long-term physiological stress load, assumed to capture the cumulative systemic exposure to glucocorticoids (90). As indicators of stress-related health, different risk (e.g., biomarkers of inflammation) or protective factors (e.g., brain-derived neurotrophic factor, BDNF) can be assessed.

#### 4.2.1 ReSource

Examining diurnal cortisol, neither total diurnal cortisol output nor the cortisol slope showed average change following ReSource mental trainings (49). We did, however, observe more subtle change depending on neural processes in that training-related structural increases in bilateral hippocampal (i.e., cornu ammonis) volume specifically after the Affect module correlated with a reduction in total diurnal cortisol output (81). There are different ways to interpret this finding. It is possible that the acceptance-based Affect training reduced the daily stress load and associated cortisol release, which, in turn, may have triggered downstream brain alterations. Alternatively, Affect training may have initially targeted cornu ammonis volume, which, as per its role as a central break of the HPA axis, improved hippocampal capacity to inhibit cortisol release (91, 92).

Irrespective of training type (Presence, Affect or Perspective), hair cortisol and cortisone concentration decreased consistently over the first 3 to 6 months of training. There was no further reduction at the final 9-month mark. These training effects on hair glucocorticoids increased with practice frequency (28). We conclude that to achieve chronic stress reduction at the level of HPA axis activation, it may be necessary to practice longer than the typical 8-week training curriculum offered in Western societies. In this study, significant improvements in subjective-psychological stress load were found in the Perceived Stress Scale [PSS; (84)] but not the Trier Inventory of Chronic Stress (TICS; 93), and for the former, selectively after the socio-cognitive Perspective training, but only in one of the three cohorts (28). We thus interpret the results with caution.

Because the immune system is implicated in numerous mental and physiological conditions including depression, cardiometabolic disease, and cancer (94, 95), MBI effects on inflammatory biomarkers are of particular interest as downstream consequences of training-induced stress reduction. The most commonly assayed biomarkers of inflammation include the pro-inflammatory cytokine interleukin-6 (IL-6), and the surrogate marker of low-grade inflammation high sensitive C-reactive protein (hs-CRP). In ReSource participants, we found no average training effect on either marker. However, examining individual differences, participants with higher inflammatory load at study baseline showed stronger reduction of IL-6, and of hs-CRP if they were male, following the Presence module (83). These findings suggest that training monitoring is most effective when targeting elevated inflammation levels, while there may be a floor effect for very healthy adults with low inflammation or stress levels.

BDNF has been identified as a key mediator in the etiology of stress-related disorders, and greater BDNF levels may be a protective factor (96, 97). Mental training that works towards stress reduction should hence increase BDNF levels. In one study, ReSource participants generally showed continuous increases in BDNF levels after 3, 6, and 9 months of training, irrespective of training type, and this effect was partially mediated by training-induced physiological stress reduction, measured via hair cortisol accumulation. Individual BDNF increases after 9-month training were further linked to simultaneous increases in hippocampal (i.e., dentate gyrus) volume (82).

In sum, findings in basal stress states are somewhat more mixed than for challenge states. Two studies suggest non-differentiable practice effects of all training types, including monitoring and acceptance, in decreasing hair cortisol/cortisone (28) and increasing BDNF levels (82). With regard to MAT’s specific predictions, only one study suggests a clear advantage of acceptance (Affect) over other training, in increasing hippocampal volume with an associated diurnal cortisol reduction. Moreover, going beyond MAT, this finding emerges irrespective of whether monitoring (Presence) is practiced before acceptance or not (81). Two findings suggest specific effects of monitoring [Presence; decreasing proinflammatory markers, (83)] or socio-cognitive training [Perspective; decreased subjective-psychological stress; (28)].

#### 4.2.2 MAT

One dismantling RCT tested MAT in a daily life ecological momentary assessment approach targeting subjective-psychological stress levels and positive/negative affect. After an 8-week group-based intervention, the authors found reduced stress (71) and negative affect ratings (72) after combined Monitor and Accept training, Monitor Only, and even after no training, suggesting retest effects. However, there was a significant advantage of combined Monitor and Accept over the two other conditions, with stronger stress reduction and an additional increase in positive affect. A similar pattern was found in a 2-week dismantling RCT showing decreases in negative affect after either combined Monitor and Accept training, Monitor Only, or an active control training (coping control program without monitoring or acceptance). Again, only combined Monitor and Accept training improved positive affect (72) (see also **Table 1**). Thus, outcomes of these comparative MAT studies relatively consistently confirm MAT, with one exception.

## 5 Summary and insights on mindfulness mechanisms of action

Although mindfulness-based interventions (MBIs) have frequently been shown to reduce psychological (dis)tress and improve wellbeing [e.g., (13, 16, 17, 98)], widely accepted theoretical frameworks of how mindfulness affects health and wellbeing remain elusive. Mechanistic concepts and understanding are hindered by commonly heterogeneous and non-granular intervention protocols that simultaneously target distinct attentional, emotional and cognitive processes. We here aimed to advance mechanistic understanding by reviewing existing comparative RCTs that specifically investigate how distinct mindfulness-based practices causally affect subjective-psychological stress and affect, as well as physiological stress and stress-related health markers. Focusing on monitoring and acceptance skills, we summarized support for differentiable effects in general, and for the monitoring and acceptance theory (MAT, 38) in particular, while separately examining effects on challenge and basal states of the stress system.

It emerges that the specific combination of monitoring and acceptance is necessary for physiological stress reduction in states of acute challenge. However, basal or more chronic physiological stress and health states benefit also from training monitoring alone. Across both states, markers of subjective-psychological stress and affect do not necessarily follow the same pattern as physiological ones, and do not follow the specific predictions of MAT. After challenge, subjective-psychological stress shows reductions irrespective of training type, and in the basal state, it follows a mixed pattern of either non-specific training effects or reductions only after combined training of monitoring and acceptance (see **Figure 1** for a graphical summary of the review results and interpretation). Evidence that training monitoring alone can have detrimental effects is limited.

Below, we discuss these findings in detail, their broader conceptual implications, and application for future training designs.

### 5.1 Evaluating MBI mechanisms of action

#### 5.1.1 MBI RCT effects on stress

First, the reviewed studies repeatedly provide evidence for reduction of both subjective-psychological and physiological stress after MBIs involving monitoring and/or acceptance (although see **Supplementary Table S1** for negative findings). Previous broader systematic reviews revealed mindfulness training effects mostly compared to passive control groups (13, 17, 98). In contrast, in the here reviewed comparative RCTs, some training-specific effects (e.g., of monitoring + acceptance) hold even when compared to other types of mindfulness-based practice (e.g., monitoring only or socio-cognitive training), which can be considered particularly well-matched active control groups. This notably strong result suggest a superiority of specific training components. Future studies should test this observation in systematic reviews of comparative RCTs, while also considering important effect moderators such as trial quality and duration.

#### 5.1.2 Monitoring and acceptance: causal evidence

**Physiological data**

Examining physiological stress markers, we find strong evidence for differential effects of training monitoring and acceptance in challenge states, but not in basal states. It appears that if the stress system is acutely activated, for example by a laboratory stressor, to ultimately facilitate physiological stress reduction, monitoring needs to be paired with an ability for acceptance. Also in line with Lindsay and Creswell (39), monitoring alone mostly leads to null-findings, but rarely has detrimental effects (cf. 99), which are only identified in one study showing a heightened cortisol awakening response (CAR) after Presence training [(49); although both high and low CAR can be considered an adaptive outcome, cf. (100)].

Typically, it is the prolonged or chronic basal stress that has the most dire health implications (1, 22). For such basal states, we find substantial evidence that training monitoring skills alone can already lead to a reduced stress load.

A possible explanation for the difference between monitoring effects in challenge versus basal states is that basal stress presumably accumulates largely from low-level stressors in daily life, for which monitoring may play a fundamentally different role than for a strong TSST-like stressor. And while acute reactivity provides a window into an individual’s overall stress load, physiological indices of acute reactivity and longer-term stress regulation are often unrelated (101), suggesting that these states may also be influenced by distinct psychosocial processes. Specifically, emotion regulation via acceptance is one of the key stress coping tools available in the constrained settings in which challenge states are typically assessed (standardized and rigid laboratory tests or automatic physiological responses as in the case of the CAR). In daily life, however, numerous alternative coping strategies may be used. For example, if monitoring training leads to a more accurate perception of daily affective states, this may trigger social support seeking or the constructive avoidance of perceived stressors.

Regarding the difference between MAT and ReSource findings on monitoring in basal states, one explanation is that the 2–8 week Monitor Only training in MAT trials was significantly shorter than 3-month monitoring (Presence) training in ReSource, possibly leading to weaker effects. For example, the above suggested mechanism from an initially improved awareness of daily strains towards adaptive behavioral coping may require several weeks to unfold. Alternatively, Lindsay and Creswell also discuss how sufficiently long, structured monitoring training by itself could begin to engender an implicit non-judgemental quality (73), thus enhancing acceptance and reducing stress. Crucially, if practiced long enough, training monitoring alone may eventually lead to stress reduction in basal states.

Across both challenge and basal states, we also find evidence that training acceptance without monitoring reduces stress. While the MAT authors suggest that acceptance should, in theory, need to be combined with monitoring to be effective (38, 39, 102), this is not a prediction formally tested in MAT protocols. Thus, we interpret any acceptance only effects as evidence that extends, but does not contradict MAT.

Further subdividing physiological outcomes, it is also notable that we find substantial evidence for changed HPA-axis, but very limited for changed SAM activity. However, only two of the examined studies actually assessed sympathetic markers (26; **Supplementary Table S1**; 73), specifically during acute challenge. Therefore, further research is needed before conclusions about differential changes in HPA and SAM axes can be drawn.

**Subjective-psychological data**

Training effects on subjective-psychological stress in acute challenge states were reported only in ReSource studies and showed no specificity to type of training, that is, all equally reduced stress. There are several potential reasons for this discrepancy to physiological markers. Subjective-psychological training effects may precede physiological ones. Alternatively, they may be driven by known biases in self-reports (103), particularly participants’ expectations in positive training outcomes (104). Irrespective of its origin, the detected lack of covariance between the subjective-psychological and physiological levels of acute stress responding is a well-known issue in stress research (19, 105).

Training effects on subjective-psychological stress perceptions and affect in basal states were reported only in MAT dismantling trials. The observed pattern of results clearly shows that as for the physiological data, MAT predictions are not reliably fulfilled in the basal state also on the subjective-psychological response level.

### 5.2 Common training mechanisms

While the present review focuses on differential effects of specific MBI training components, it is important to note that non-specific aspects of MBIs, such as social interaction, group dynamics, participant expectations and instructor support, can also significantly contribute to MBI effects on stress related health outcomes. Practice intensity [e.g., hours of class practice; (28), but see also (36)] and intervention duration are additional general effect moderators, and, as indicated above, some health outcomes may take months of intense practice to materialize (28, 106). Comparative trainings are designed to control for the influence of common factors across conditions, and the here reviewed evidence demonstrates differential training effects (e.g., of monitoring versus acceptance) above and beyond the influence of common factors. Nonetheless, to improve MBI efficacy and effectiveness, some researchers have argued for a stronger focus on these common factors (107), rather than taking a more granular approach of differential training mechanism, as is suggested here.

### 5.3 Recommendations for future interventions

Mechanistic understanding of MBI effects is an important step towards personalizing intervention programs to different populations, contexts and needs. For the design of future MBIs and based on the insights of this review, it may be effective to customize intervention protocols to individual stress-regulation goals. For example, individuals facing acute psychosocial challenges in their profession may benefit most from training acceptance alone or in combination with monitoring. For dealing with more continuous low-level stressors of daily life, training monitoring may be an equally good fit. Given that monitoring and acceptance appear to have positive yet distinct effects, and their combined training often produces the strongest effects especially on psychological stress and affect, combined training of monitoring and acceptance is nonetheless recommendable. To achieve longer term physiological stress reduction and improve downstream stress-related health, particular attention should be paid to training routines that can be maintained for at least several weeks to months.

The effectiveness of MBIs and their specific components may also differ based on practitioners’ characteristics and between populations (108). For example, higher neuroticism prior to training has been associated with greater improvement in psychological distress post training (109). Similarly, training acceptance may be a particularly effective strategy to improve suboptimal emotion regulation skills in practitioners with higher levels of neuroticism (110). Nonetheless, current evidence for individual-difference moderators of MBI effectiveness remains limited (17), and personalizing MBIs to individuals and populations continues to be an important challenge for future work that crucially requires much larger samples (111). Particular attention should be paid to customizing interventions to individuals at greater risk for developing stress-related disorders, such as those exposed to early life stress (112).

Studies aiming to further advance mechanistic understanding should also assess the physiological pathways through which MBIs affect stress-related health in more detail. For example, increased activity in stress regulatory brain regions such as the prefrontal cortex, and decreased activity in stress reactive regions such as the amygdala have been proposed as the two central pathways of MBI physical health outcomes (30). Future comparative RCTs should examine alterations in neural activity and connectivity of these regions in relation to stress-related health outcomes. Moreover, the present review highlights important differences between challenge and basal stress states. In everyday life, however, stress levels fluctuate dynamically between them. Monitoring stress with a higher temporal resolution, for example through the use of rapidly developing wearable cortisol sensors [e.g., (113)], is a promising avenue to capture individual diurnal stress dynamics more accurately, and subsequently develop individualized just-in-time adaptive interventions (114).

### 5.4 Limitations

The conclusions that can be drawn from this selective review are subject to several limitations. While the ReSource modules Presence and Affect map well onto MAT, their training involves several components that go beyond monitoring and acceptance training. It therefore cannot be precluded that stress reduction after Affect, for example, was driven by increased positive affect or compassion, rather than acceptance (alone). Relatedly, evaluating Resource only regarding monitoring and acceptance components does not do justice to the full complexity the training.

Moreover, the three comparative RCTs examined here focused on healthy adults. As such, they primarily give insights to stress-related health mechanisms in healthy populations and for the purpose of disorder prevention. Future research may investigate whether the same mechanistic distinctions hold in clinical and at-risk samples.

The present work presents an important first step towards conceptually validating and extending MAT’s predictions with evidence from an independent investigation, the lack of which has previously been criticized (102). Nevertheless, more data from additional comparative trials is needed to corroborate the identified pattern.

## 6 Conclusion

A mechanistic understanding of the effects of mindfulness-based interventions is crucial for advancing the field of contemplative science. This review of mindfulness-based comparative RCTs suggests that successful reduction of the psycho-physiological stress load depends on both the type of intervention, specifically the training of monitoring versus acceptance, and the type of outcome, specifically psychological and physiological markers of challenge versus basal stress states. Overall, the reviewed studies corroborate and extend predictions of the Monitor and Acceptance Theory with evidence from the independent ReSource Project. In particular, it emerges that training acceptance alone, and monitoring alone in basal states, can already reduce physiological stress activity. Future studies may further investigate the unique benefits of acceptance, compared to contexts where successful monitoring is a prerequisite for stress regulation.

## Footnotes

1 Given that overload with psychosocial stressors is the predominant cause of chronic stress in modern societies (23), not feeling stressed or mounting relatively lower physiological stress responses to psychosocial challenges in the first place can be considered the most adaptive response [next to a timely downregulation, see e.g., (24)]. The same argument cannot be made for patients with blunted HPA axis reactivity (e.g., with depression or autoimmune disorders), who tend to release too little stress-reactive cortisol despite high levels of subjective stress (25).
