# Supplementary material for: How mindfulness-based training improves stress-related health: a selective review of randomized clinical trials comparing psychological mechanisms of action
Source: Front Endocrinol (Lausanne). 2025 Aug 4;16:1415081. doi: 10.3389/fendo.2025.1415081 (PMC12358272; doi:10.3389/fendo.2025.1415081)
Supplement: Supplementary file 1 [file Table1.docx]

***Table S1.*** Summary of null findings stemming from randomized controlled studies comparing different mechanisms of mindfulness-based interventions.

| **Challenge states** | | | |
| --- | --- | --- | --- |
| **RCT** | **Study** | **Method** | **Null finding** |
|  |  |  | **Stress marker** |
| ReSource (N=332) | Engert et al., 2017 | 3-6 months intervention  TSST | Alpha-amylase, heart rate, high frequency heart rate variability, C-reactive protein, and interleukin-6 reactivity |
| Dismantling Trial 2 (N=153) | Lindsay et al., 2018 | 2-week intervention  TSST-like | Subjective-psychological reactivity (feelings of stress, anxiousness, and insecurity on visual analogue scale from 0-100) |
| **Basal states** | | | |
| ReSource (N=332) | Linz et al., 2022^†^ | 3-9 months intervention  Daily life sampling | Subjective-psychological stress (stress intensity on visual analogue scale from 1-20), affect (pleasure-displeasure on scale from 1-9), cortisol levels |
|  | Engert et al., 2023 | 3-9 months intervention  Daily life sampling | Cortisol slope over the course of the day, total daily cortisol output |
|  | Puhlmann et al., 2021 | 3-9 months intervention  Hair sampling | Subjective-psychological stress (TICS; PSS showed selective training effects after the Perspective module) |

Notes: PSS: Perceived Stress Scale (Cohen et al., 1983); TICS: Trier Inventory for Chronic Stress (Schulz, P., & Schlotz, 1999).

^†^As additional variables of interest, outside the domain of subjective-psychological stress and affect, we here measured arousal, coping efficacy, and thought patterns, all of which showed training-related change.

**Supplementary References**

Cohen, S., Kamarck, T., & Mermelstein, R. (1983). A Global Measure of Perceived Stress. *Journal of Health and Social Behavior*, *24*(4), 385–396.

Engert, V., Kok, B. E., Papassotiriou, I., Chrousos, G. P., & Singer, T. (2017). Specific reduction in cortisol stress reactivity after social but not attention-based mental training. *Science Advances*, *3*, 1–13.

Engert, V., Hoehne, K., & Singer, T. (2023). Specific Reduction in the Cortisol Awakening Response after Socio-Affective Mental Training. *Mindfulness*, 681–694. <https://doi.org/10.1007/s12671-023-02074-y>

Lindsay, E. K., Young, S., Smyth, J. M., Brown, K. W., & Creswell, J. D. (2018). Acceptance lowers stress reactivity: Dismantling mindfulness training in a randomized controlled trial. *Psychoneuroendocrinology*, *87*, 63–73. https://doi.org/10.1016/j.psyneuen.2017.09.015

Linz, R., Puhlmann, L. M. C., Engert, V., & Singer, T. (2022). Investigating the impact of distinct contemplative mental trainings on daily life stress, thoughts and affect—Evidence from a nine-month longitudinal ecological momentary assessment study. *Psychoneuroendocrinology*, *142*(March), 105800. <https://doi.org/10.1016/j.psyneuen.2022.105800>

Puhlmann, L. M. C., Vrtička, P., Linz, R., Stalder, T., Kirschbaum, C., Engert, V., & Singer, T. (2021). Contemplative Mental Training Reduces Hair Glucocorticoid Levels in a Randomized Clinical Trial. *Psychosomatic Medicine*, *83*(8), 894–905. <https://doi.org/10.1097/PSY.0000000000000970>

Schulz, P., & Schlotz, W. (1999). Trierer Inventar zur Erfassung von Chronischem Stress (TICS): Skalenkonstruktion, teststatistische Überprüfung und Validierung der Skala Arbeitsüberlastung. *Diagnostica*, *45*, 8–19.
